# Calcium Intake in Elderly Australian Women Is Inadequate

**DOI:** 10.3390/nu2091036

**Published:** 2010-09-17

**Authors:** Xingqiong Meng, Deborah A. Kerr, Kun Zhu, Amanda Devine, Vicky Solah, Colin W. Binns, Richard L. Prince

**Affiliations:** 1 Curtin Health Innovation Research Institute and the School of Public Health, Curtin University of Technology, Perth, WA 6845, Australia; Email: RosieMeng@cancerqld.org.au (X.M.); v.solah@curtin.edu.au (V.S.); c.binns@curtin.edu.au (C.W.B.); 2 Department of Endocrinology and Diabetes, Sir Charles Gairdner Hospital, Nedlands, WA 6009, Australia; Email: kzhu@meddent.uwa.edu.au (K.Z.); richard.prince@uwa.edu.au (R.L.P.); 3 School of Medicine and Pharmacology, University of Western Australia, Crawley, WA 6009, Australia; 4 School of Exercise, Biomedical and Health Sciences, Edith Cowan University, Joondalup, WA 6027, Australia; Email: a.devine@ecu.edu.au

**Keywords:** calcium intake, elderly women, mineral and vitamin supplement

## Abstract

The role of calcium in the prevention of bone loss in later life has been well established but little data exist on the adequacy of calcium intakes in elderly Australian women. The aim of this study was to compare the dietary intake including calcium of elderly Australian women with the Australian dietary recommendation, and to investigate the prevalence of calcium supplement use in this population. Community-dwelling women aged 70–80 years were randomly recruited using the Electoral Roll for a 2-year protein intervention study in Western Australia. Dietary intake was assessed at baseline by a 3-day weighed food record and analysed for energy, calcium and other nutrients. A total of 218 women were included in the analysis. Mean energy intake was 7,140 ± 1,518 kJ/day and protein provided 19 ± 4% of energy. Mean dietary calcium intake was 852 ± 298 mg/day, which is below Australian recommendations. Less than one quarter of women reported taking calcium supplements and only 3% reported taking vitamin D supplements. Calcium supplements by average provided calcium 122 ± 427 mg/day and when this was taken into account, total calcium intake increased to 955 ± 504 mg/day, which remained 13% lower than the Estimated Average Requirement (EAR, 1,100 mg/day) for women of this age group. The women taking calcium supplements had a higher calcium intake (1501 ± 573 mg) compared with the women on diet alone (813 ± 347 mg). The results of this study indicate that the majority of elderly women were not meeting their calcium requirements from diet alone. In order to achieve the recommended dietary calcium intake, better strategies for promoting increased calcium, from both diet and calcium supplements appears to be needed.

## 1. Introduction

Osteoporosis and related fracture represent a significant public health burden globally. Factors affecting bone mass include genetic, endocrine and lifestyle factors, which includes nutrition. Evidence from previous studies has shown the effectiveness of calcium supplementation or calcium combined with vitamin D supplementation in preventing osteoporotic fracture in elderly women [1,2]. However, poor adherence appears to be a major factor limiting the efficacy of calcium and vitamin D supplementation [3,4]. A five-year longitudinal calcium intervention study in elderly Australian women showed that the long term compliance to supplements was poor [2], as only 58% of women in the calcium supplemented group and 56% of women in the placebo group actually took 80% or more of their pills. A nationwide survey in 9,851 postmenopausal women referred to 141 Italian centres for osteoporosis management stated that a lack of motivation was given as the most frequent reason for discontinuation of calcium and/or vitamin D supplementation [5]. Therefore, due to the likely poor compliance with supplements, guidelines for ensuring an adequate calcium intake from diet become increasingly important. 

There are very little data on how much calcium elderly women obtain from their diet, what contribution calcium supplements make to their total calcium intake, and if they achieved the current Australian recommendation. To the best of our knowledge, little data exist on the dietary patterns and use of dietary supplements, including calcium, in elderly Australian women. To date, the Australia National Nutrition Survey (NNS) in 1995 [6] was the only nationwide assessment of dietary intakes in Australian adults. The average intake of calcium for women over 65 was 685 mg/day, which is about two-third of the Estimated Average Requirement (EAR) and half of the Recommended Dietary Intake (RDI) for calcium for this age group. The EAR and RDI for calcium for postmenopausal women are 1,100 mg/day and 1,300 mg/day, respectively [7]. The EAR is a daily nutrient level estimated to meet the requirements of half the healthy individuals in a particular life stage and gender group. It is used to estimate the prevalence of inadequate intakes in groups. The RDI is the average daily dietary intake level that is sufficient to meet the nutrient requirements of nearly all (97–98 percent) healthy individuals in a particular life stage and gender group. 

Since the 1995 National Nutrition Survey, organizations such as Osteoporosis Australia have been working hard to promote the role of calcium in preventing Osteoporosis, but there are no data indicating if there has been a shift in intake over this time. Therefore, the aim of this study was to compare the dietary intake of elderly Australian women with the RDIs and EARs for calcium and other nutrients, and to examine the prevalence of vitamin and mineral supplements use in this population. 

## 2. Materials and Methods

### 2.1. Subjects

Two hundred and nineteen ambulant community-dwelling women aged between 70 and 80 years were recruited during April and September 2007 for a two-year randomised controlled dietary protein intervention trial. Letters were sent to 2,356 individuals selected randomly from the Electoral Roll, which has the names and addresses of all women of this age range who were registered to vote. Since voting is compulsory in Australia this is the most complete population information available which ensured a population based study. A total of 837 (36% of letters sent) women responded, and 254 (30% of the respondents) women were eligible to attend the clinic screening. After the screening visit, 36 subjects were found to be ineligible for the study and were excluded. The study was approved by the Human Research Ethics Committees of Sir Charles Gardiner Hospital and Curtin University and informed consent was obtained from each participant. Subjects were excluded if they had bone disease (apart from osteoporosis); were taking medication for osteoporosis (Bisphosphonates, Evista, Teriparatide, Protos, hormone replacement treatment) apart from calcium or vitamin D; had a protein intake higher than 1.5 g/kg body weight per day; had cognitive impairment (Mini mental state exam <2.4); body mass index (BMI) >35 kg/m^2^; had bowel surgery resulting in malabsorption or other conditions; Coeliac disease; clinical hepatic insufficiency; clinical diagnosis of diabetes and renal insufficiency. 

### 2.2. Dietary intake assessment

Dietary intake was assessed by a 3-day weighed food record. The participants were asked to record everything they ate and drank for three consecutive days which included two week days and one weekend day (Thursday, Friday and Saturday, or Sunday, Monday and Tuesday). They watched a training video on how to complete their food record and they were also provided with electronic food scales (Philips, HR 2385/A, Hungary). They were instructed not to alter what they ate or drank during this time and to record as accurately as they could using either the food scales provided or household measures (for example cups and spoon measures). On the day they returned the food record, the participant was interviewed by a trained research assistant to clarify types and amount of food recorded. In addition, details of any vitamin and mineral supplements consumed in the 3-day period were recorded. These data were coded and analysed by nutritionists who had completed advanced competency training in dietary assessment. The food record was analysed for calcium, energy and other nutrients intakes using the AusNut 2007 database (Foodworks Professional edition version 3.02, Xyris, QLD). The energy intake was calculated with dietary fibre. 

### 2.3. Anthropometry

Anthropometric measurements including height and weight were taken in the morning of clinic visit while the subjects were wearing a light hospital gown. The measurements were made following the standard protocol of the International Standards for Anthropometric Assessment [8]. Standing height was measured using a wall-mounted stadiometer (Veeder-Root, Elizabethtown, NC, USA) to the nearest 0.1 cm. Body weight was measured using an electronic scale (August Sauter GmbH D-7470 Albstadt 1 Ebingen, West Germany) to the nearest 0.1 kg. 

### 2.4. Statistical analysis

Statistical analyses were made using SPSS version 17.0 (Chicago, IL, USA) and Excel 2007. Descriptive analysis method was used. The homogeneity of variance and normality assumptions were checked by Levene and Kolmogorov-Smirnov tests.

## 3. Results and Discussion

### 3.1. Dietary energy and macronutrient intakes

A total of 218 subjects were included in the analysis as one of the enrolled subjects failed to return her 3-day food record. The mean age of participants was 74 ± 3 years and BMI was 26.8 ± 3.9 kg/m^2^categorising the women as slightly overweight (Table 1). The mean energy intake was 7084 ± 1462 kJ/d, which is greater than that reported in the 1995 NNS (Table 2). The mean protein intake was 75 ± 17 g/day (1.14 ± 0.33 g/kg/day), higher than 64 g/day reported in the 1995 NNS, and greater than the EAR of 46 g/d (0.75 g/kg body weight/day) and the RDI of 57 g/day (0.94 g/kg body weight/day) for women aged over 70 years. The proportions of energy obtained from protein, fat and carbohydrate in the current study were similar to those estimated in the 1995 NNS and were within the acceptable macronutrient distribution range for preventing chronic disease [7]. 

**Table 1:** Characteristics of participants in the current study (n = 218) and in the National Nutrition Survey (NNS) in 1995, Australia.

	Current study	1995 NNS
	(Mean ± SD)	(Mean)
Age (years)	74.2 ± 2.7	65 and over
Weight (kg)	68.5 ± 11.3	66.1
Height (cm)	159.9 ± 6.0	156.7
BMI (kg/m^2^)	26.8 ± 3.9	26.9

**Table 2:** Mean dietary intakes in the current study (n = 218) compared to the 1995 National Nutritional Survey (NNS) and Australian recommendations.

	Current study	1995 NNS	EAR/AMDR
	age 74.2 ± 2.7 years	for age ≥65 years	for age >70 years
	(Mean ± SD)	(Mean)	(Mean)
Energy (kJ)	7084 ± 1462	6367	8573 EER ^1^
Protein (g)	75 ± 17	64	46
Protein (g/kg body weight)	1.13 ± 0.31	NA	0.75
Fat (g)	62 ± 19	57	NA
Carbohydrate (g)	187 ± 44	182	NA
Alcohol (g)	7 ± 10	5	NA
Energy contribution of macronutrients			
% energy from protein	19 ± 3	18	15–25
% energy from fat	33 ± 6	32	20–35
% energy from carbohydrate	46 ± 7	48	45–65
% energy from alcohol	3 ± 4	2	NA

EAR: Estimated Average Requirement; AMDR: Acceptable Macronutrient Distribution Range; NA: not available; EER ^1^: Estimated Energy Requirement calculated from mean height 1.59 m, mean weight of 68.5 kg and light physical activity level.

### 3.2. Calcium intake

Mean dietary calcium intake from food alone was 852 ± 298 mg/day, which was 19% higher than the 1995 NNS, but remains 23% lower than the EAR for calcium intake of 1,100 mg/day for postmenopausal women (Table 3). Although dietary calcium intakes were higher for the present study compared to the 1995 NNS, since different methodologies were used, technically a direct comparison cannot be made. The current study used a 3-day weighed food record whereas a 24-hour recall was used in the 1995 NNS which might not account for the daily variation in calcium intake. Another possible reason for a higher intake of calcium in the current study compared with the 1995 NNS could be because the subjects in the present study may be more “health aware”, which may not be representative of all community-dwelling women of this age range. Moreover, the differences in the study population between the current study and the 1995 NNS may also contribute to the difference in calcium intake. Firstly, the age range of the study population is slightly different (women aged 70–80 years in the current study *versus* women aged 65 and above in the 1995 NNS). Secondly, the geographical base is different (Western Australia for the current study *versus* whole of Australia in the 1995 NNS). 

**Table 3:** Mean dietary mineral intake in the current study (n = 218) compared to the 1995 National Nutritional Survey (NNS) and Australian recommendations.

	Current study	1995 NNS	EAR
	age 74.2 ± 2.7 years	age ≥65 years	for age >70 years
	(Mean ± SD)	(Mean)	(Mean)
Calcium (mg)	852 ± 298	686	1100
Phosphorus (mg)	1343 ± 323	1132	580
Magnesium (mg)	299 ± 74	268	265
Iron (mg)	11 ± 3	11	5
Zinc (mg)	10 ± 2	9	6.5
Potassium (mg)	2973 ± 649	2626	2800 (AI)

EAR: Estimated Average Requirement; AI: Adequate intake (used when RDI cannot be determined).

At a population level, there is evidence that an increase in calcium intake may have occurred. The overall per capital milk consumption has been increasing in Australia over the past five years [9]. This increase in consumption appears to be attributable to an increase in the consumption of reduced fat milk as whole milk has remained relatively stable [10]. A national survey using the same methodology (24-hour recall) as the 1995 NNS is clearly needed to confirm whether there has been a shift in the calcium intake at a population level. 

In a population based calcium intervention study we conducted in 1998, the baseline dietary calcium intake of 862 women aged 70–80 years was 966 ± 349 mg/day, which is higher than the dietary calcium intake in the present study [11]. The use of a self-administered food-frequency questionnaire (FFQ) in the 1998 study may contribute to the higher value because FFQ tends to overestimate average absolute nutrient intakes [12]. Dietary intakes of other minerals reported in this study population were similar to those in the 1995 survey and exceeded the respective recommended intake levels (Table 3). 

**Figure 1:**
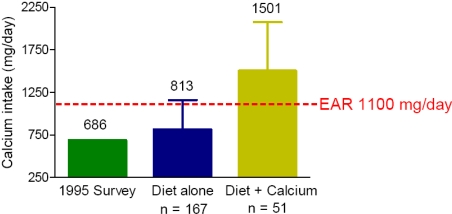
Comparison of calcium intake in the current study participants with intake estimated in the 1995 National Nutrition Survey, with the Estimated Average Requirement (EAR).

The mean and standard deviation of calcium intake including calcium intake from supplements was 955 ± 504 mg/day in the current study (Figure 1). The average calcium intake from supplements alone was 122 ± 427 mg/day, which indicates that the amount of calcium from supplements is only adding a small amount (13%) to the total calcium intake and diet remains the major calcium source. Only 23% of subjects were taking calcium supplements and 3% taking vitamin D supplements in the present study (Table 4). As shown in Figure 1, the women who were taking calcium supplements had a higher calcium intake (1501 ± 573 mg) compared with the women on diet alone (813 ± 347 mg). There are few data on the types of supplements that elderly Australian women are taking. Australia’s National Nutrition Survey in 1995 showed that 12% of all those aged 65–74 years reported the use of herbal or natural products in the 2 weeks before the survey, however these products were not defined. Brownie conducted a survey in 1,200 (49% females) elderly Australians aged 65–98 years in 2000 [13]. Among 662 females, only 9.6% of subjects reported taking calcium supplements. The prevalence of calcium supplementation (23%) was higher in the present study compared to Brownie’s study [13]. This may be due to a difference in study subject recruitment including demographic and time difference. In the current study subjects were recruited for a two year randomized controlled trial of protein supplementation and represent a motivated group of “well” elderly and many therefore may represent a “best of” population where dietary intakes are concerned, whereas the NNS was a population based survey sampling across a range of demographics and socioeconomic status. However, this could also be due to an increase in calcium supplement use with the increased awareness of the health benefit of calcium. Either way the majority of elderly Australian women are not meeting the recommended dietary intake for calcium as was also the case 12 years ago. Because of the low rate and poor compliance of using calcium supplements in this age group of women, it is difficult to see that simply promoting a higher dose of calcium would ensure they are meeting their calcium requirements, without addressing the barriers to taking calcium supplement in the community. 

**Table 4:** Prevalence of supplements use in study participants (n = 218).

Supplements	Number of subjects using supplements (%)
Calcium	51 (23)
Vitamin D	6 (3)
Fish oil	55 (25)
Other vitamins or minerals	43 (20)

## 4. Conclusions

The results of this study indicate that elderly women are not meeting their calcium requirements from diet alone. Women taking calcium supplements have intakes above the estimated average requirement but only 23% were taking calcium supplements. At a population level, promoting calcium-fortified foods, such as calcium-fortified bread or rice, may be an effective strategy to increasing calcium intake and needs to further explored. In order to achieve the recommended dietary calcium intake, better strategies for promoting increased dietary calcium, together with calcium supplementation appears to be needed. 

## Declare

We declare that we have no conflict of interest.
